# Human Metapneumovirus RNA in Encephalitis Patient

**DOI:** 10.3201/eid1103.040676

**Published:** 2005-03

**Authors:** Oliver Schildgen, Thomas Glatzel, Tilman Geikowski, Bärbel Scheibner, Arne Simon, Lutz Bindl, Mark Born, Sergei Viazov, Anja Wilkesmann, Gisela Knöpfle, Michael Roggendorf, Bertfried Matz

**Affiliations:** * University of Bonn, Bonn, Germany; † University Hospital Essen, Essen, Germany; 1 These authors contributed equally to this study.

**Keywords:** human metapneumovirus, fatal encephalitis, dispatch

## Abstract

We describe a fatal case of encephalitis that might be correlated with primary human metapneumovirus (HMPV) encephalitis. Postmortem HMPV RNA was detected in brain and lung tissue samples from the patient. Furthermore, HMPV RNA was found in culture fluids from cells coincubated with lung tissue.

Human metapneumovirus (HMPV), a new member of the paramyxovirus family closely related to respiratory syncytial virus (RSV), was first described in 2001 (*1*). Subsequent reports demonstrated that HMPV is distributed worldwide (*2*–*5*), causing mild-to-severe acute infections of the nasopharyngeal tract (*6*,*7*). Although neurologic symptoms have been described for infections caused by other paramyxoviruses, such as Hendra, Nipah, mumps, and measles (*8*–*12*), no such symptoms have been associated with HMPV infection. We describe a child who died from edema caused by encephalitis probably induced or triggered by HMPV.

## The Case

A 14-month-old boy unresponsive to verbal and tactile stimulation with high fever (temperature 39°C) was admitted to a primary care hospital ≈30 minutes after the onset of generalized febrile convulsions that did not respond to 2 doses of rectal diazepam (5 mg). His weight was 8 kg, all extremities were warm and well perfused, his heart rate was 140 beats per minute (bpm), and oxygen saturation was 93% in ambient air. Initial clinical examination showed no purpuric or petechial lesions, no heart murmurs or pulmonary rales, and no palpable enlargement of the liver or spleen. The boy had been healthy until 2 days earlier, when rhinorrhea, mild pharyngitis, and cough developed without signs of lower respiratory tract involvement. On the day of admission, he vomited once in the morning and than refused to drink. In the afternoon of the same day, a high fever developed, and seizures began, with extension and struggling of all extremities but without opisthotonos. The patient initially turned both eyes upwards but later stared straight ahead with no spontaneous eye movements and fixed pupils of 3 to 4 mm in diameter.

The boy had been born at 34 weeks’ gestational age (weight 2,300 g), and he was treated for a few days with antimicrobial drugs after prolonged rupture of amniotic sac membranes, even though early-onset infection had not been confirmed. His neonatal period was uneventful, with the exception of moderate withdrawal symptoms (irritability, frequent bowel movements) probably due to tobacco use by his mother during pregnancy. The primary pediatrician documented a subtle, generalized muscular hypotension at the age of 9 months but did not consider this finding remarkable enough to investigate further (sonographic examination of the central nervous system showed normal results immediately after birth and 2 weeks later).

Clinical seizure activity subsided ≈2 hours after the intravenous administration of diazepam, clonazepam, phenobarbital, and lorazepam. Blood pressure was stable without high volume infusion or vasosuppressor support, and oxygen saturation (pulse oximetry) was 100% with 2 L of supplemental oxygen. Glasgow coma scale (GCS) was 10 at the end of the seizure. Cerebrospinal fluid (CSF) drawn by lumbar puncture showed no pleocytosis, glucose level of 4.6 mmol/L, and a slightly elevated protein content of 116 mg/dL (normal value <45mg/dL) (Table 1). Although leukocyte count and serum C-reactive protein did not suggest inflammation, empiric antimicrobial chemotherapy with ceftriaxone, ampicillin, gentamicin, and acyclovir was started immediately. An electroencephalogram showed generalized slow waves but no seizure activity.

**Table 1:** Overview of laboratory parameters tested before the patient was admitted to the primary care hospital

Parameter	Measured	Reference range
Hemoglobin	11.2 g/dL	11–14.4 g/dL
Thrombocytes	264/mm^3^	286–509/mm^3^
Leukocytes	5,700/mm^3^	6,000–17,500/mm^3^
Neutrophils	47%	55%–75%
Band forms	8%	2%–8%
Lymphocytes	37%	20%–40%
Monocytes	7%	0%–12%
Eosinophils	1%	0%–7%
C-reactive protein	1 mg/L	0–3 mg/L
Ammonium	43 μmol/L (<94)	15–55 μmol/L

**Figure 1:**
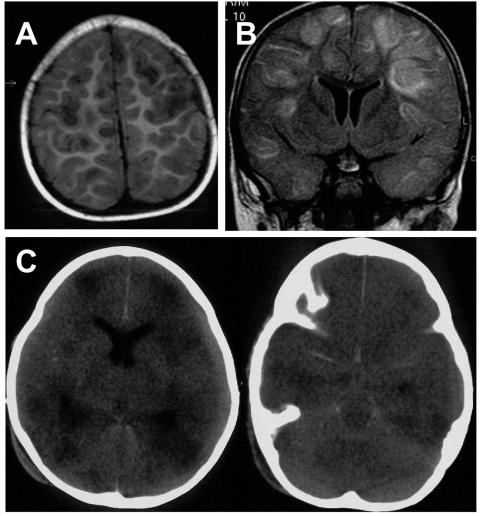
Axial T1-weighted magnetic resonance imaging (MRI) scan (A) and coronal fluid attenuated inversion recovery (FLAIR) (B) show multifocal, mainly cortical and subcortical lesions of high signal intensity, which are most probably caused by multifocal encephalitis. C) Nonenhanced axial computed tomographic (CT) scan performed 2 days after the MRI shows multiple, hypodense lesions and signs of general edema. Additionally, it shows a hyperdense arachnoid collection that was not yet visible on the MRI 2 days before (panels A and B).

On the basis of abnormal findings on a magnetic resonance imaging scan (MRI) performed 12 hours after admission (Figure 1A and 1B), attending physicians diagnosed meningoencephalitis of unknown origin. During the next few hours, the GCS of the patient deteriorated to 5. Both pupils were 8 mm in diameter and not reactive to light. The patient was intubated (no gag reflex observed during the procedure) and mechanically ventilated. He was sent to the pediatric intensive care unit of a tertiary-care hospital 24 hours after admission to have an external ventricular drain inserted. Upon arrival, the patient was deeply comatose (GCS 5) without response to painful stimuli; he had rectal temperature of 32°C, arrhythmic heart rate (90 bpm), and a mean arterial pressure of 29 mm Hg.

Corneal reflexes and gag reflex were silent. No spontaneous movements were observed; pupils were 5 mm in diameter and fixed. A chest radiograph excluded a pneumonic infection and confirmed the correct position of the ventilation tube and the central venous catheter tip. After stabilization of vital signs, the patient’s condition did not change. The abnormal findings on a computed (CT) tomography scan are shown in Figure 1C. Intracranial pressure measured after inserting an external ventricular drain and repeatedly afterwards was constantly elevated to 90 cm H_2_O. Brain tissue extruded locally, and the drain was removed after 2 days. Repeated cultures of blood, urine, CSF, brain tissue, and the tip of the external drainage did not show any bacterial (culture) or viral pathogen (culture and polymerase chain reaction [PCR]) (Table 2). The patient did not exclusively display symptoms typical of measles or mumps, and neither of these viruses could be isolated from brain tissue.

**Table 2:** Virologic tests performed on patient specimens*†

Virus	CSF	Brain	Liver	Spleen	Kidney	Lung	Heart	Diagnostic methods	Tested before death?
HSV (PCR)	–	–	–	ND	–	–	–	In-house PCR (nested), cell culture	Yes (from CSF and brain biopsy)
VZV (PCR)	–	–	ND	ND	ND	–	ND	In-house PCR (nested), cell culture	Yes (from CSF and brain biopsy)
Adenovirus (PCR)		–	–	–	–	–	–	In-house PCR (nested)	–
HHV-6 (PCR)		–	–	–	–	–	–	In-house PCR (nested)	–
HBV (PCR)		–	–	–	–	–	–	In-house PCR (nested)	–
HCV (PCR)		–	–	–	–	–	–	In-house PCR (nested)	–
ParvoB19 (PCR)		–	–	–	–	–	–	In-house PCR (nested)	–
CMV (PCR)		–	–	–	–	–	–	In-house PCR (nested), cell culture	–
Enterovirus (PCR)		–	–	–	ND	–	–	In-house RT-PCR, cell culture	–
RSV (Antigen)		ND	ND	ND	ND	–	ND	Antigen ELISA, Directigen RSV (Becton Dickinison, Heidelberg, Germany)	–
RSV (cell culture)		–	–	–	–	–	–	Cell culture	–
Influenza A+B		–	–	–	–	–	–	Cell culture	–
Mumps		–	–	–	–	–	–	Cell culture	Measles excluded, no symptoms of mumps†
Measles		–	–	–	–	–	–	Cell culture	
HMPV (PCR)	–	+	ND	ND	ND	+	ND	In-house RT-PCR (*5*,*13*)	–
HMPV (PCR from cell culture supernatant)	–	+	ND	ND	ND	+	ND	In-house RT-PCR (*5*,*13*) from Vero-cell culture supernatant	–

*CSF, cerebrospinal fluid; HSV, herpes simplex virus; PCR, polymerase chain reaction; ND, not done; VZV, varicella–zoster virus; HHV, human herpesvirus; HBV, hepatitis B virus; HCV, hepatitis C virus; CMV, cytomegalovirus; RT, reverse transcriptase; RSV, respiratory syncytial virus; ELISA, enzyme-linked immunosorbent assay; HMPV, human metapneumovirus. †Before death, viral encephalitis induced by HSV and VZV was excluded by nested PCR from the CSF and also from a biopsy. Measles and mumps were excluded by the primary anamnestic protocol (no exanthema or other clinical symptoms). The anamnestic protocol also showed a successful vaccination against those pathogens. Furthermore, no clinical symptoms of mumps were observed. Postmortem specimens from brain, lung, kidney, liver, spleen, and heart were mounted on different cell lines. Detailed PCR protocols are available on request.

In addition, extensive acute investigations of serum and urine specimens did not confirm any underlying inborn or acquired metabolic illness. A battered-child syndrome was excluded by a normal ophthalmologic examination and radiographs of all extremities; no sign of fresh or old fractures was found. After 10 days of supportive intensive care without clinical improvement, in accordance to the current regulations of German federal law, the child was considered to be dead and extubated after receiving informed consent from his legal guardian. He died shortly thereafter, and an autopsy was performed.

## Conclusions

The acute symptoms were managed sufficiently by the primary intensive care team, particularly in terms of oxygenation, and no signs of dehydration were seen at admission. Thus, extensive discussion with the attending intensive care, neurosurgery, neuroradiology, and pediatric neurology team led to the conclusion that the time course of the illness suggested encephalitis as the primary reason for the child’s symptoms and the adverse outcome. Other differential diagnoses of the MRI and CT findings such as postictal edema after a status epilepticus, edema due to prolonged hyperpyrexia and dehydration, or hypoxemia were considered highly improbable.

Tissue specimens from brain, lung, liver, kidney, and heart as well as serum and CSF were intensively screened for bacterial and viral infections by using standard techniques (Table 2). No bacterial or viral pathogens, except HMPV, were detected. RNA of this virus was identified by reverse transcription (RT)–PCR in both brain and lung tissue specimens. Moreover, all tissue specimens were mounted routinely on different cell lines susceptible to viruses commonly known to cause encephalitis (Table 3), but HMPV RNA was detected by PCR only in the cell culture supernatant of a Vero culture mounted with lung tissue for 3 weeks. Although no cytopathic effect was observed in any of the cell cultures, the fluid from the Vero cells mounted with lung tissue was repeatedly positive for HMPV RNA. None of the other viruses was detected by PCR, and no cytopathic effect was seen in any of the cell cultures.

**Table 3:** Cell lines routinely used for isolation of individual viral pathogens*†

Cell line	HSV	VZV	CMV	RSV	Mumps	Measles	Enterovirus	Influenza	HMPV
Vero	+	–		–	+	+	+	–	+
LLC-MK2	–	–	–	–	–	–	–	–	+
MS	–	–	–	+	–	–	–	–	+
MDCK	–	–	–	–	–	–	–	+	–
RD	–	–	–	–	–	–	+	–	–
HEF	+	+	+	–	–	–	–	–	–

*The + symbol indicates that the cell line is susceptible to the virus and was used for diagnostic procedures. †HSV, herpes simplex virus; VZV, varicella zoster virus; CMV, cytomegalovirus; RSV, respiratory syncytial virus; HMPV, human metapneumovirus; LLC-MK2, kidney cell line from rhesus monkey; MS, monkey stable; MDCK, Madin-Darbin canine kidney; RD, human Caucasian rhabdomyosarcoma; HEF, human embryonic fibroblasts.

**Figure 2:**
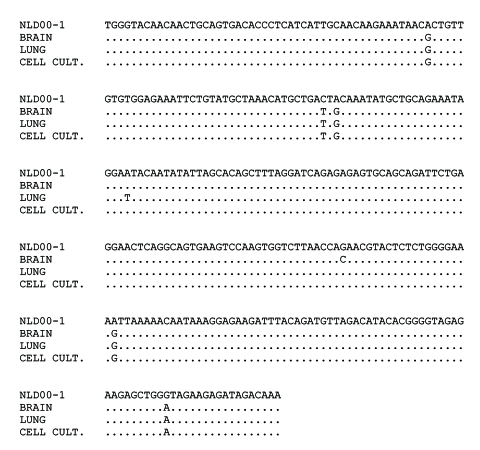
Alignment of 302 nucleotide human metapneumovirus (HMPV) sequences amplified from brain and lung tissues of the patient and from the supernatant of infected Vero cell culture. Sequence of the HMPV strain NLD00-1 was used as a prototype sequence. Conditions of the reverse transcription–polymerase chain reaction were described elsewhere (*5*).

Amplified fragments of HMPV DNA derived from the Vero cell culture supernatant were subjected to direct sequencing. The alignment of the resulting sequences showed close relatedness of HMPV RNA sequences present in brain and lung tissues and those obtained from the supernatant of infected cell culture (Figure 2). Upon phylogenetic analysis, the identified HMPV sequences were clearly separated from the sequences of other HMPV isolates obtained by our laboratory (*13*). These results allowed us to exclude the possibility of contamination of the brain and lung tissues under investigation with HMPV sequences from another source.

Virologic data on the detection of HMPV were complemented by results of histologic and immunochemical investigations. Thus, both the lung and the brain tissues showed evidence of an active inflammation. The alveolar lumina were partly or completely filled with fluid mixed with inflammatory cells, and the alveolar walls were thickened. The alveoli in half of the specimens were atelectatic. Some bronchioles had segmental loss of epithelium; meninges were diffusely thickened by many neutrophils and a few macrophages mixed with fibrin and a few erythrocytes.

Unfortunately, specific immune staining of paraffin-embedded tissues did not allow us to detect HMPV antigens in the brain or lung of the patient. Several reasons may explain these negative results, including low concentration of viral antigens in the infected tissues, relatively low sensitivity of the immunochemical procedures, and a high sensitivity of the RT-PCR assay. Similar observations may be noted, namely, the positive RT-PCR findings of viral RNA and the inflammatory response in investigated tissues in the case of Nipah virus infection of the brain (*11*).

To our knowledge, this case report is the first of fatal encephalitis that might be associated with HMPV infection. We base a possible etiologic relationship between HMPV and the observed neurologic manifestations on the detection of HMPV RNA in the brain and lung tissues. Some clinical observations might serve as circumstantial evidence to support this hypothesis. The clinical course and MRI data for our patient are very similar to those observed in several patients with fatal encephalitis associated with Nipah virus infection, another member of the paramyxovirus family (*8*,*11*). Definite conclusions on the possible involvement of HMPV in neurologic disorders might be drawn only after additional studies. At this stage, however, we recommend HMPV screening for patients, especially young children, with symptoms of encephalitis of unknown origin. These investigations might extend our knowledge of the clinical manifestations and consequences of HMPV infection.
